# A Green Approach for High Oxidation Resistance, Flexible Transparent Conductive Films Based on Reduced Graphene Oxide and Copper Nanowires

**DOI:** 10.1186/s11671-022-03716-1

**Published:** 2022-08-24

**Authors:** Ya-Ting Lin, Da-Wei Huang, Pin-Feng Huang, Li-Chun Chang, Yi-Ting Lai, Nyan-Hwa Tai

**Affiliations:** 1 Department of Materials Science and Engineering, National Tsing Hua University, Hsinchu, 30013 Taiwan; 2 Department of Materials Engineering, Ming Chi University of Technology, New Taipei City, 24301 Taiwan; 3 Center for Plasma and Thin Film Technologies, Ming Chi University of Technology, New Taipei City, Taiwan; 4 Biochemical Technology R&D Center, Ming Chi University of Technology, New Taipei City, Taiwan

## Abstract

Copper nanowires (CuNWs)-based thin film is one of the potential alternatives to tin-doped indium oxide (ITO) in terms of transparent conductive films (TCFs). However, the severe problem of atmospheric oxidation restricts their practical applications. In this work, we develop a simple approach to fabricate highly stable TCFs through the dip-coating method using reduced graphene oxide (rGO) and CuNWs as the primary materials. Compared with previous works using toxic reduction agents, herein, the CuNWs are synthesized via a green aqueous process using glucose and lactic acid as the reductants, and rGO is prepared through the modified Hummers’ method followed by a hydrogen-annealing process to form hydrogen-annealing-reduced graphene oxide (h-rGO). In the rGO/CuNWs films, the dip-coated graphene oxide layer can increase the adhesion of the CuNWs on the substrate, and the fabricated h-rGO/CuNWs can exhibit high atmospheric oxidation resistance and excellent flexibility. The sheet resistance of the h-rGO/CuNWs film only increased from 25.1 to 42.2 Ω/sq after exposure to ambient atmosphere for 30 days and remained almost unchanged after the dynamic bending test for 2500 cycles at a constant radius of 5.3 mm. The h-rGO/CuNWs TCF can be not only fabricated via a route with a superior inexpensive and safe method but also possessed competitive optoelectronic properties with high electrical stability and flexibility, demonstrating great opportunities for future optoelectronic applications.

**Supplementary Information:**

The online version contains supplementary material available at 10.1186/s11671-022-03716-1.

## Introduction

Transparent conductive films (TCFs), which are responsible for electric conduction and light transmission, play a crucial role in various optoelectronic devices, such as displays [1–3], solar cells [4, 5], electrochromic devices [6, 7], and transparent film heaters [8, 9]. Tin-doped indium oxide (ITO) is currently the most popular material for TCFs due to its excellent transmittance (*T* ≈ 90% @ 550 nm) and low sheet resistance (*R*_sh_ ≈ 20 Ω/sq) [10]. However, ITO possesses some drawbacks, such as brittleness, the rising cost of indium (increased by more than 80% to 240 USD/kilogram from 2020 to 2022, according to TRADING ECONOMICS [11]), and the requirement for high vacuum during deposition, which limit its applications in large-scale flexible optoelectronic devices [12]. In this regard, various materials, such as carbon nanotubes (CNTs) [13, 14], graphene-based materials [15, 16], conducting polymers [17, 18], and metal nanowires [19–22], have been proposed as substitutes for ITO to achieve flexible devices with better performance.

Graphene-based materials have been considered promising materials for application in flexible devices because of their good mechanical properties, high optical transparency, and prominent thermal and electrical properties [23–25]. Although chemical vapor deposition (CVD) enables the growth and transfer of carbon nanomaterials to various substrates and thus can achieve excellent electrical and optical properties comparable to those of ITO [26], the high energy consumption of the CVD process and inevitable damage during film transfer hinder its industrial applications, which require an economical route and scalable product [1, 27]. Compared with graphene prepared through CVD, reduced graphene oxide (rGO) has been extensively utilized to achieve mass production of large-area flexible TCFs [28, 29]. Typically, the preparation of rGO adopts reduction techniques to eliminate the oxygen-containing functional groups on graphene oxide (GO) [30], leading to the restoration of the sp^2^-bonded graphene with recovered conductivity [31]. Reduction methods include utilization of chemical reagents and annealing at high temperature. However, the widely used chemical reductants for GO, such as hydrazine [32] and hydriodic acid (HI) [31], are highly toxic to humans and harmful to the environment. High-temperature thermal reduction is an effective alternative method to prepare rGO [33], but it can only be used for specific substrates that can withstand high temperatures. In addition, the reduction of GO is inevitably associated with low graphitization, numerous defects, and favoring restacking with disorder arrangement, resulting in a decrease in electrical properties and nonuniform rGO distribution on TCFs [20, 34, 35].

Metal nanowires have recently received considerable attention not only for their high transmittance and electrical conductivity but also for their excellent mechanical flexibility, triggering calls for studying the feasibility of metal nanowires to replace ITO-based TCFs [8]. Unfortunately, the applicability of metal nanowires is challenging due to poor adhesion on the substrate, high roughness, and atmospheric oxidation [36]. In addition, the poor contacts at wire-wire junctions and large open areas in the partially percolated metal nanowire network become obstacles to realize metal nanowires-based TCFs with high optoelectronic performance and long-term durability [35]. Major effort has been made by some pioneering works, which used silver nanowires (AgNWs) covered by protectively conductive layers to not only improve the adhesion and roughness but also fill the vacancies between wires and protect AgNWs from oxidation [20, 37–39]. However, the cost of AgNWs-based TCFs is concerning, restricting its scalable and sustainable application. In this regard, copper possesses high electric conductivity, which is only approximately 5% less than that of silver but is 100 times cheaper and 1000 times more abundant on earth [40], which emerges as a potential substitute for fabricating commercial flexible TCFs. Although recent progress has been proposed for synthesizing CuNWs, the high toxicity of reducing agents [41], the time-consuming process (> 12 h) [42], and the relatively high temperature (approximately 120 °C) [43, 44] limit the practical applications of CuNWs in the concern of environmental sustainability.

Herein, we developed a simple and cost-effective route for the fabrication of CuNWs-based TCFs in which the ease of oxidation can be suppressed by uniformly coating the rGO layers. The CuNWs were synthesized through a solution-based method using glucose as a green reducing agent, followed by vacuum filtration and transfer processes for preparing flexible CuNWs TCFs. The uniform coating of rGO layers onto the flexible CuNWs film can not only cover the void space formed by entangled CuNWs to provide local and long-range conductivity but also prevent the CuNWs from undergoing atmospheric oxidation. In addition, two synthesis methods, chemically reduced GO (c-rGO) and hydrogen-annealing reduced GO (h-rGO), for the preparation of rGO were adopted to investigate the long-term optoelectronic performance of the rGO/CuNWs TCFs. The h-rGO/CuNWs achieved high optoelectronic properties with a sheet resistance (*R*_sh_) of 25.1 Ω/sq and a transmittance of 85.88%, and the *R*_sh_ only slightly increased to 42.0 Ω/sq after exposure to ambient atmosphere for 30 days, exhibiting excellent resistance to atmospheric corrosion. The figure of merit (FOM) value is widely used to evaluate the optimal compromise between the optical transmittance and electrical conductivity of materials, in which a higher FOM value indicates more promising optoelectronic performance. In this work, the FOM of h-rGO/CuNWs TCFs can reach a value of 142.77, and *R*_sh_ only increased by 1.0 Ω/sq after dynamic bending tests for 2500 cycles, which is comparable to other state-of-the-art flexible TCFs.

## Experimental

### Materials

Graphite flake (325 mesh), potassium permanganate (KMnO_4_), and sodium borohydride (NaBH_4_) were purchased from Alfa Aesar. Copper chloride dehydrate (CuCl_2_ · 2H_2_O), hexadecylamine (HDA), lactic acid, sulfuric acid (H_2_SO_4_), and hydrogen peroxide (H_2_O_2_) were purchased from Sigma-Aldrich. D^+^-glucose (glucose) was purchased from SHOWA Chemical. All chemicals were used without further treatment.

### Synthesis of CuNWs

**Fig. 1:**
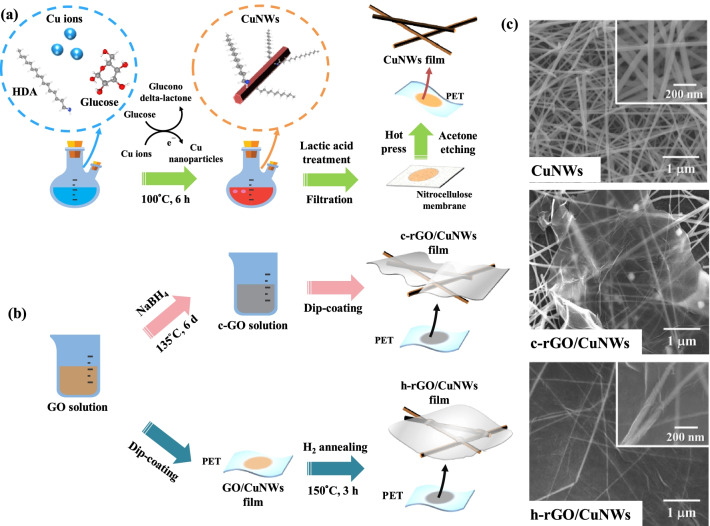
**a** Schematic illustration of the preparation of CuNWs film, which used glucose as a reducing agent and HDA as a capping agent and adopted lactic acid to remove residual copper oxide and other organic chemicals. **b** Schematic illustration of the preparation of c-rGO/CuNWs and h-rGO/CuNWs films. **c** FESEM images of CuNWs, c-rGO/CuNWs, and h-rGO/NWs. The CuNWs exhibit an average diameter of 57.78 ± 9.18 nm and a length of > 10 μm. The CuNWs network cannot be completely covered by c-rGO layers, leading to invalid protection. In contrast, h-rGO can uniformly cover the CuNWs and increase the contact area between CuNWs

To synthesize CuNWs, CuCl_2_ 2H_2_O (0.21 g), glucose (0.5 g), and HDA (1.35 g) were dissolved in 100 ml of DI water in a two-necked round-bottom flask, which was magnetically stirred at room temperature overnight to obtain a well-dispersed blue solution (Fig. 1a). The solution was then heated to 100 °C under stirring in an oil bath for 6 h. The reddish brown solution obtained from the reaction was then washed with isopropyl alcohol (IPA) and hexane in a centrifuge several times to remove the excess reactant. Finally, the purified CuNWs were stored in IPA under vacuum. In the subsequent tests, a concentration of 30.5 ppm CuNWs solution was adopted, and the measurement was taken on an inductively coupled plasma optical emission spectrometer.

### Preparation of CuNWs TCFs

CuNWs films were prepared by vacuum filtration and then transferred to polyethylene terephthalate (PET) substrates. In short, a CuNWs solution with a concentration of 30.5 ppm was sonicated for a few seconds to obtain a well-dispersed mixture. Lactic acid was added to the CuNWs dispersion for 30 s to eliminate the copper oxide and some residual organics. Subsequently, a specific volume of CuNWs solution was filtered through a nitrocellulose membrane with an average pore size of 0.45 μm. The density of the CuNWs was modulated by adjusting the filtrate volume from 3 to 12 ml. The nitrocellulose membrane covered with CuNW film was then transferred onto a PET substrate and subsequently subjected to hot pressing under uniform pressure of 15.7 MPa at 34 °C for 5 min, followed by immersion in acetone to remove the nitrocellulose membrane and subsequently drying under vacuum to obtain CuNWs TCFs.

### Preparation of GO and c-rGO

GO was synthesized according to the modified Hummers’ method. Briefly, 1.0 g of natural graphite powder was added to 100 ml of concentrated H_2_SO_4_ solution, which was stirred in an ice bath for 1 h. Subsequently, 4.0 g of KMnO_4_ was gradually added to the solution, followed by stirring at room temperature for 6 h. Then, 100 ml of DI water and 40 ml of 30% H_2_O_2_ were gradually added to the solution under stirring, and the color of the mixture changed from brown to brilliant yellow. The solution was kept at room temperature overnight, and the precipitate was collected and washed with DI water until neutralization using centrifugation. The obtained GO was added to IPA followed by sonication for 1 h to obtain a GO dispersion.

The reducing agent NaBH_4_ (4 g) was added to 500 ml GO solution followed by stirring at 135 °C for 6 days. After the reaction was completed, the alkaline solution was washed repeatedly until neutralization and subsequently dried to obtain c-rGO powder.

### Preparation of c-rGO/CuNWs and h-rGO/CuNWs films

To fabricate the rGO/CuNWs films, c-rGO powder (0.1 g) was dispersed in 10 ml of IPA solution and sonicated for 10 min to obtain a well-dispersed mixture. As shown in Fig. 1b, the c-rGO/CuNWs film was prepared through the dip-coating method by immersing the CuNWs TCF into the c-rGO mixture for 10 s followed by drying at 50 °C on a hot plate to remove the IPA solvent. The dip-coating process was repeated for various times to investigate the optoelectronic properties of c-rGO/CuNWs TCFs with different c-rGO contents.

For the preparation of the h-rGO/CuNWs film, the CuNWs TCF was immersed in the GO solution (1 mg/ml) using the same dip-coating process as that of c-rGO/CuNWs film to obtain GO/CuNWs film. The film was then annealed at 150 °C in a H_2_ atmosphere to obtain the h-rGO/CuNWs films (Fig. 1b).

### Characteristics

The surface morphologies of the samples were characterized by field emission scanning electron microscopy (FESEM, JEOL JSM-6500F). The surface roughness of the sample was characterized by atomic force microscope (AFM, Dimension Edge, Bruker) using the tapping mode. The crystal structures were verified by X-ray diffraction (XRD, Shimadzu XRD6000). A Raman spectroscope (Horiba Jobin Yvon LABRAM HR 800 UV) equipped with a 514-nm Ar^+^ laser was used to analyze the bonding structure of the prepared TCFs. The *R*_sh_ of the TCF films was measured by a two-probe method in which the probes were positioned on two parallel Ag-ink electrodes with a coplanar square configuration. The optical transmittance was measured on a UV–Vis–NIR spectrophotometer (U-4100, Hitachi). The flexibility of TCF films at a radius of 5.3 mm for 2500 bending cycles was tested on a laboratory-made bending tester.

## Results and Discussion

A pioneering work regarding the growth of CuNWs has been reported by Xie’s group [45]; in their work, CuNWs were synthesized through a hydrothermal method using glucose as a green reducing agent and HDA as a capping agent. As shown in Fig. 1a, glucose can be oxidized to glucono delta-lactone, reducing the copper ions to form copper nanoparticles via aggregation [46]. During the reaction, HDA binds preferentially onto the (100) facets of Cu and thereby enables Cu to grow nanowires along the orientation of ⟨110⟩. Figure 1c shows FESEM images of the pristine CuNWs, c-rGO/CuNWs, and h-rGO/CuNWs films. The high aspect ratio of CuNWs, with an average diameter of 57.78 ± 9.18 nm and a length of > 10 μm, allows them to form continuous conducting paths for electron transfer and multiple empty spaces for light transmittance. After the CuNWs films were dipped into a solution containing the c-rGO dispersion, the CuNWs films were covered with c-rGO. However, the wrinkled c-rGO layers reveal imperfect covering on CuNWs due to the difficulty of dispersion as well as the aggregation of rGO in nature [23]. In contrast, the h-rGO layers exhibit good coverage on CuNWs film compared with the c-rGO/CuNWs film (the SEM image with low magnification of h-rGO/CuNWs is shown in Additional file 1: Fig. S1). As mentioned, h-rGO/CuNW films were derived by dipping CuNWs films in a GO dispersion and subsequent annealing under a hydrogen atmosphere. Various functional groups on the GO surface enable great dispersity, facilitating tight and uniform coverage onto CuNWs films; thereby, after the hydrogen reduction process, h-rGO can act as an air barrier for better oxidation resistance.

Before filtration and transferring processes, the CuNWs dispersion was treated with lactic acid to etch away the adsorbed HDA and surface copper oxide/hydroxide according to the following equations [47]:

1 $$\mathrm{Cu}{\left(\mathrm{OH}\right)}_{2}+2\left[{\mathrm{CH}}_{3}\mathrm{CH}\left(\mathrm{OH}\right)\mathrm{COOH}\right]\to \mathrm{Cu}{[{\mathrm{CH}}_{3}\mathrm{CH}(\mathrm{OH})\mathrm{COO}]}_{2}+{\mathrm{H}}_{2}\mathrm{O}$$

2 $$\mathrm{CuO}+2\left[{\mathrm{CH}}_{3}\mathrm{CH}\left(\mathrm{OH}\right)\mathrm{COOH}\right]\to \mathrm{Cu}{[{\mathrm{CH}}_{3}\mathrm{CH}(\mathrm{OH})\mathrm{COO}]}_{2}+{\mathrm{H}}_{2}\mathrm{O}$$

**Fig. 2:**
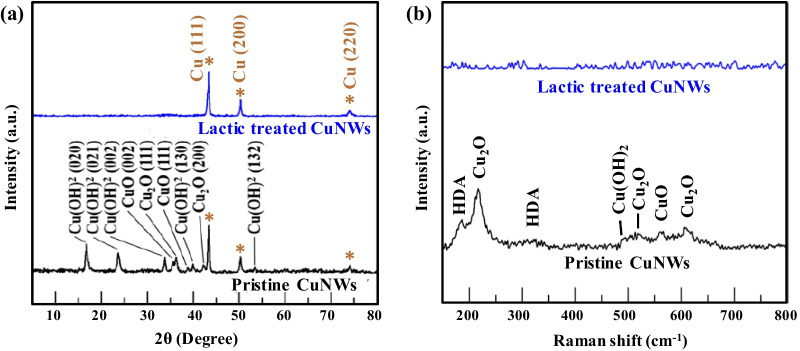
**a** XRD patterns of the CuNWs before and after lactic acid treatment. The intensive peaks correspond to the (111), (200), and (220) crystal planes of the face-centered cubic structure of Cu, and no extra signals appear after the lactic acid treatment, indicating the high purity of the CuNWs. **b** Raman spectra of CuNWs before and after lactic acid treatment. After treatment with lactic acid, the surface HDA and oxides/hydroxides were removed

Therefore, lactic acid can react with copper oxide/hydroxide and form copper lactate, which is soluble in solvent and can be readily washed away. The XRD results confirm that the copper oxide/hydroxide was removed by lactic acid treatment (Fig. 2a). After lactic acid treatment, three obvious peaks at 43.5°, 50.7°, and 74.48° attributed to the copper crystal plane of (111), (200), and (220), respectively, are observed, indicating that CuNWs have a face-centered cubic structure (JCPDS 03-1018). Moreover, other peaks arising from the presence of copper oxide/hydroxides disappear, confirming that the lactic acid treatment resulted in high-purity CuNWs. In Fig. 2b, the Raman spectra also support the hypothesis that the oxide/hydroxide and HDA surfaces were removed after lactic acid treatment. The characteristic Raman peaks at approximately 218/523/623 cm^−1^ and 580 cm^−1^ are attributed to the Cu_2_O and CuO phases, respectively [48, 49]. Cu(OH)_2_ shows a weak characteristic peak at about 490 cm^−1^ [49]. In addition, a visible peak at ~ 180 cm^−1^ along with a wide and weak peak at 340 cm^−1^ could be assigned to acoustical motions of alkane chains in HDA [50]. All the above-mentioned characteristic bands in the pristine CuNWs disappeared after lactic acid treatment, indicating the removal of HDA and copper oxide/hydroxide; hence, electrons can transport between CuNWs network with less resistance.

**Fig. 3:**
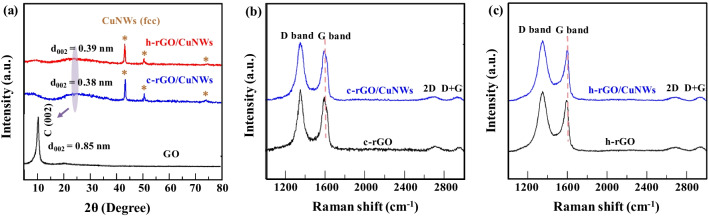
**a** XRD patterns of GO, c-rGO/CuNWs, and h-rGO/CuNWs films. The graphitic peak (002) shifts to higher angles after chemical reduction and hydrogen-annealing reduction, indicating the restacking of rGO sheets. The copper crystal planes show the rGO-coated CuNWs films. **b** Raman spectra of c-rGO and c-rGO/CuNWs films. **c** Raman spectra of h-rGO and h-rGO/CuNWs films

As depicted in Fig. 3a, the XRD pattern of GO reveals a dominant peak of the graphitic (002) plane at 2*θ* = 10.4° [51], corresponding to a layer d-spacing of 0.85 nm, which is shifted to 23.6° (d-spacing = 0.38 nm) and 23.4° (d-spacing = 0.39 nm) after chemical reduction by NaBH_4_ and hydrogen-annealing reduction, respectively. The disappearance of the broad (002) diffraction peak and a decrease in interlayer distance indicate restacking of the rGO sheets due to the removal of oxygen-containing functional groups during the reduction. Furthermore, the (111), (200) and (220) diffraction peaks attributed to the pure Cu phase were detected (as depicted by the brown star signs in Fig. 3a) in c-rGO/CuNWs and h-rGO/CuNWs films, implying that coating with c-rGO or h-rGO insignificantly affects the structure of the CuNWs.

Raman spectroscopy was used to examine the structure of GO as well as the coating of rGO layers on CuNWs (as summarized in Additional file 1: Table S1). In Additional file 1: Fig. S2, GO exhibits two prominent peaks; the D band indicates disorder of the graphitic structure or defects, and the G bands refer to sp^2^-bonded carbon atoms, which appear at 1351 and 1603 cm^−1^, respectively. The characteristic peaks obtained from Raman spectroscopy are consistent with a previously reported article regarding carbon materials [52]. Herein, the intensity ratio of the D and G bands (*I*_D_/*I*_G_ ratio) increases from 0.99 for GO to 1.19 and 1.18 for c-rGO and h-rGO, respectively. The increase in defects caused by the removal of the oxygen-containing functional groups on GO hence verifies the formation of rGO. In addition, the weak 2D and D + G bands at 2600–3000 cm^−1^, arising from the sp^2^ domains and the π band in the graphitic electronic structure, can be used to define the graphene layers by peak shift and peak shape [53]. Based upon the peak positions in c-rGO and h-rGO, the 2D and D + G bands are approximately 2695 cm^−1^ and 2920 cm^−1^, respectively, confirming the disordered structure of multilayered graphene (2–10 layers) [54]. Comparing c-rGO with h-rGO, the Raman results of the c-rGO/CuNWs and h-rGO/CuNWs films, depicted in Fig. 3b and c, respectively, show the same I_D_/I_G_ ratio, suggesting that the degrees of reduction for c-rGO and h-rGO are not affected after coating on the CuNWs film. Notably, the upshift of the G band from 1593(c-rGO/CuNWs) to 1998 cm^−1^ (c-rGO) can be observed; likewise, the upshift of the G band from 1590 cm^−1^ (h-rGO/CuNWs) to 1600 cm^−1^ (h-rGO) is also detected. The higher upshifting of the G band indicates a stronger p-type doping effect of h-rGO/CuNWs, which probably originated from a strong interaction existing in the rGO–Cu interface. The interfacial interaction may result from the chemical bonding between Cu and functional groups on GO, leading to not only a high interfacial adhesion but also an enlarged work function difference at the interface between rGO and Cu, which is beneficial for charge transfer across the rGO–Cu interface [55, 56]; therefore, after the H_2_-annealing reduction, a facilitated charge transfer process can be expected in h-rGO/CuNWs network, which will be beneficial for TCF applications.

**Fig. 4:**
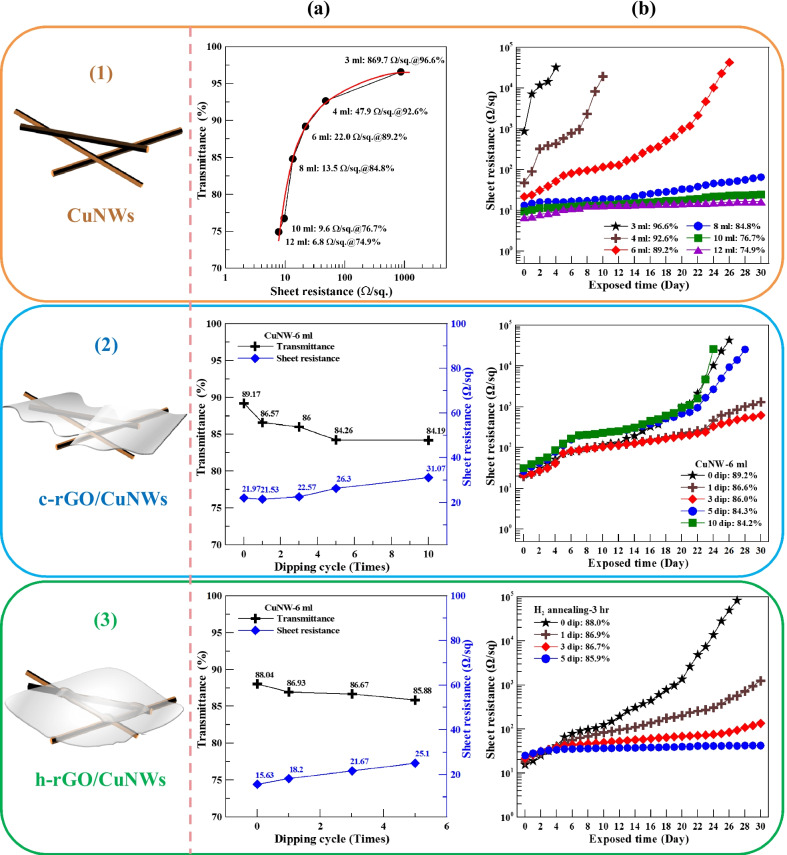
Schematic illustration of (1) CuNWs, (2) c-rGO/CuNWs films, and (3) h-rGO/CuNWs films. Column **a** optical transmittance versus sheet resistance and **b** variations of the sheet resistance versus exposed time

Herein, we also studied the optoelectronic properties (*T* at λ = 550 nm) as well as the resistance to atmospheric oxidation of the pristine CuNWs, c-rGO/CuNWs, and h-rGO/CuNWs TCFs. As depicted in Fig. 4(1a), both *T* and *R*_sh_ of CuNWs film decrease as the CuNWs density increases, where the density was designed by controlling the vacuum filtration amount of the CuNWs solution. The optoelectronic properties of R_sh_ and *T* change from 869.7 Ω/sq and 96.6% to values of 6.8 Ω/sq and 74.9%, respectively, as the amount of the CuNWs solution increases from 3 to 12 ml. Moreover, there is a turning point at *R*_sh_ = 22.0 Ω/sq and *T* = 89.2% when 6 ml of the CuNWs solution was used, indicating a desirable optoelectronic performance with low *R*_sh_ and high *T*; therefore, 6 ml of the CuNWs solution was adopted in the subsequent studies. The oxidation resistance of the CuNWs TCFs was investigated by measuring the change in R_sh_ after different exposure durations to ambient atmosphere. In Fig. 4(1b), owing to the ease of oxidation of the CuNWs film, R_sh_ increases dramatically as the CuNWs density is low (3 and 4 ml CuNWs solution), implying that the sparse electrical conductive path is easily destroyed by oxidation. It is also noted that the CuNWs film with high density (> 8 ml CuNWs solution) shows good electrical stability because the dense CuNWs network can serve as a barrier to protect the CuNWs located at the bottom from oxidation, providing a conductive path for the electrical percolation threshold. However, the sacrifice of transmittance (*T* < 80%) makes the CuNWs films impracticable for optoelectronic applications.

Therefore, a transparent gas barrier to prevent oxidation of the CuNWs film is imperative. In this regard, the CuNWs films (6 ml CuNWs solution) were coated with c-rGO and h-rGO, and their optoelectronic performance and oxidation resistance were investigated. As shown in Figs. 4(2a) and (3a), both the c-rGO/CuNWs and the h-rGO/CuNWs films exhibited the same trend of increasing R_sh_ and decreasing *T* as the number of dipping cycles increased. Although an increase in the rGO layers reduces the local resistance, the overall sheet resistance of the TCF is dominated by the CuNWs; therefore, excessive rGO layers suppress electron transport in the rGO/CuNWs films. It is worth noting that the *R*_sh_ value of the c-rGO/CuNWs film with high dipping cycles (> 5 times) shows a dramatic increase after 20 days, as shown in Fig. 4(2b), which could be attributed to crevice corrosion induced by the low oxygen concentration in the gap between wrinkled c-rGO and CuNWs [57]. Copper oxidation is an electrochemical reaction involving the transport of electrons to the cathode. The primary anodic and cathodic reactions of copper oxidation are described by Eqs. (3) and (4), respectively [58]:

3 $$\mathrm{Cu }\to {\mathrm{Cu}}^{2+}+2{\mathrm{e}}^{-}$$

4 $$\frac{1}{2}{\mathrm{O}}_{2}+{\mathrm{H}}_{2}\mathrm{O}+2{\mathrm{e}}^{-}\to 2{\mathrm{OH}}^{-}$$

**Fig. 5:**
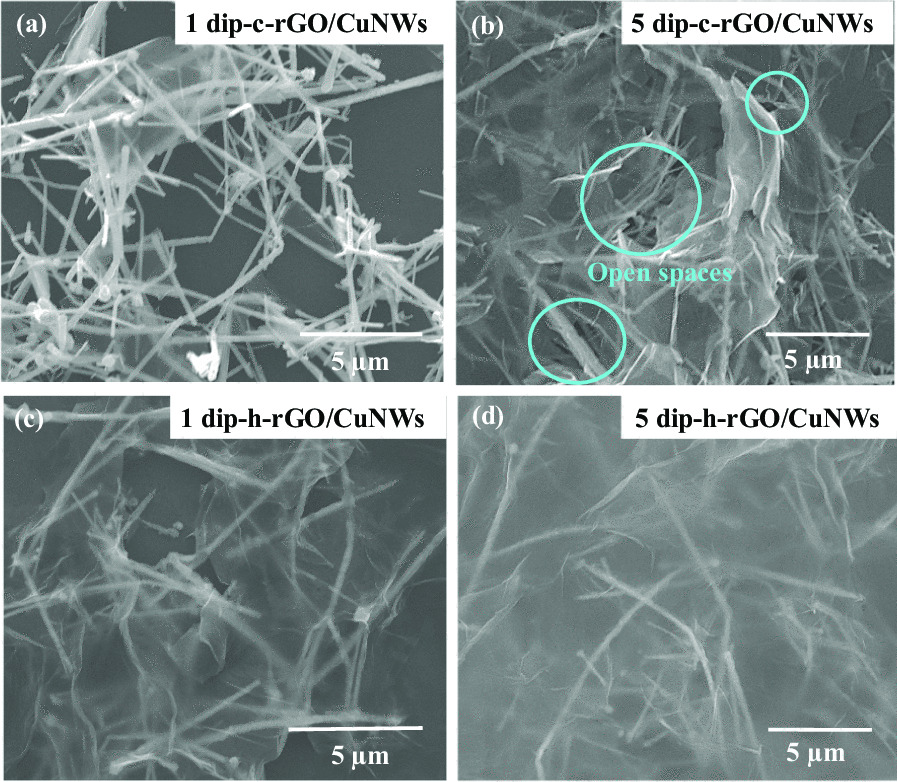
FESEM images of c-rGO/CuNWs with **a** 1 dipping cycle and **b** 5 dipping cycles. The open spaces between nonuniform c-rGO layers result in accelerated corrosion of CuNWs. FESEM images of h-rGO/CuNWs with **c** 1 dipping cycle and **d** 5 dipping cycles. The h-rGO/CuNWs with more dipping cycles shows large-area and uniform coverage of h-rGO layers on CuNWs surface

As the dipping cycle increases, the nonuniform c-rGO distribution and non-adhesion c-rGO layers result in various open spaces at c-rGO/CuNWs surface, as shown in Fig. 5a and b. Because the crevices between c-rGO and CuNWs merely have extremely low oxygen concentrations, it leads to local anodic oxidation, while cathodic reduction occurs at the rest of the material, which results in accelerated corrosion. In contrast, increasing dipping cycles enable h-rGO layers to uniform cover the overall CuNWs film (Fig. 5c and d). Additional file 1: Fig S3a and S3b show the AFM image of h-rGO/CuNWs film and the line profile along the blue dashed lines in the AFM image, respectively. The h-rGO layers can cover the overall CuNWs surface with surface roughness average (Ra) and root mean square roughness (Rq) of 5.4 nm and 7.2 nm, respectively. The step height between two r-GO sheets is approximately 3.4 nm, indicating a three-layered rGO structure. In addition, the h-rGO/CuNWs TCF with 5 dipping cycles shows long-term durability after 30 days, which only results in a slight increase in *R*_sh_ from 25.1 to 42.2 Ω/sq while retaining an acceptable transmittance of 85.9%. The stable atmospheric oxidation resistance of h-rGO/CuNWs TCFs demonstrates that h-rGO can uniformly cover the CuNWs and play a role in atmospheric corrosion protection.

**Fig. 6:**
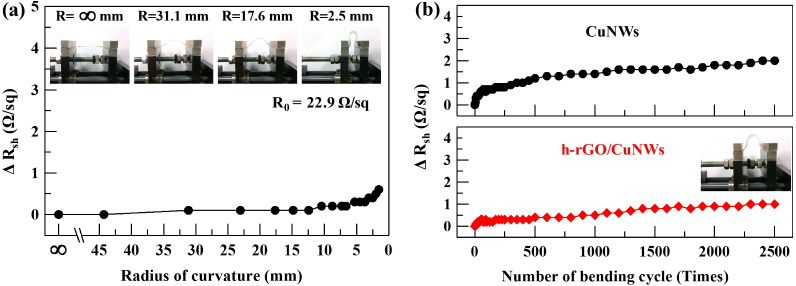
**a** Sheet resistance as a function of the radius of curvature of the h-rGO/CuNWs film. **b** Variation in the sheet resistance of the CuNWs and h-rGO/CuNWs films on the PET substrate as a function of bending cycles. The films were bent to a radius of curvature of 5.3 mm, as shown in the inset

We conducted dynamic bending tests to examine the flexibility of the CuNWs and h-rGO/CuNWs TCFs. In Fig. 6a, no obvious increase in sheet resistance of the h-rGO/CuNWs TCF was detected as the sample was bent to a radius of 5.0 mm. Even though the radius of curvature was further decreased to 1.6 mm, the *R*_sh_ of h-rGO/CuNW TCFs showed only a negligible increase of 0.6 Ω/sq, implying the excellent flexibility of the h-rGO/CuNWs TCFs compared with ITO. Moreover, the CuNWs and the h-rGO/CuNWs TCFs were bent for 2,500 cycles under a bending radius of 5.3 mm to test the electromechanical stabilities; as shown in Fig. 6b, both the CuNWs and the h-rGO/CuNWs TCFs showed good flexibility with Δ*R*_sh_ values of 2.0 and 1.0 Ω/sq, respectively. The superior flexibility of the h-rGO/CuNWs TCFs is contributed by the tight adhesion between the h-rGO and CuNWs, which stabilizes the CuNWs on the substrate.

**Fig. 7:**
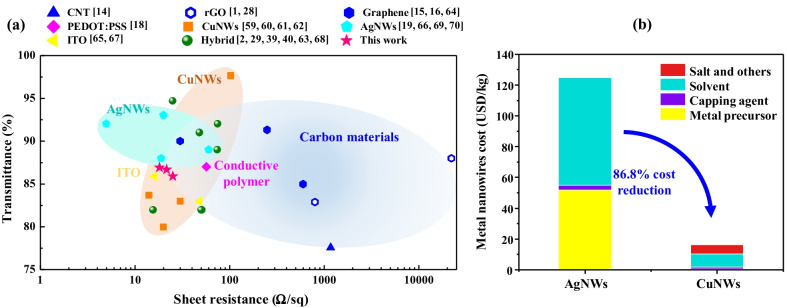
**a** Optoelectronic performances of selected state-of-the-art flexible TCFs. **b** Price analysis of preparation of the CuNWs and AgNWs. The total price is divided into four parts (metal precursor, capping agent, solvent, salt and other chemicals), which clearly show that the fabrication of CuNWs is more cost-effective

The FOM value is generally used to evaluate the optimal compromise between the optical transmittance and electrical conductivity of materials. The FOM can be calculated according to the equations shown as follows [59–61]:

5 $$T = \left( {{ }1 + { }\frac{188.5}{{R_{{{\text{sh}}}} }}\frac{{\sigma_{{{\text{OP}}}} }}{{\sigma_{{{\text{DC}}}} }}} \right)^{ - 2}$$

6 $${\text{FOM}} = { }\frac{{\sigma_{{{\text{OP}}}} }}{{\sigma_{{{\text{DC}}}} }} = \frac{188.5}{{R_{{{\text{sh}}}} \left( {\frac{1}{\sqrt T } - 1} \right)}}$$

where *T* is the transmittance, ${R}_{\mathrm{sh}}$ is the sheet resistance, *σ*_OP_ is the optical conductivity and *σ*_DC_ _*is*_ the direct current conductivity. The higher FOM values indicate lower sheet resistance at a given transmittance. To provide a holistic optoelectronic performance, we summarize the transmittance, sheet resistance, and FOM value of selected state-of-the-art flexible TCFs, as shown in Fig. 6a and Additional file 1: Table S1 in the Supporting Information [1, 2, 14–16, 18, 19, 28, 29, 39, 40, 59–70]. The optoelectronic performance of h-rGO/CuNWs TCF achieves *R*_sh_ = 18.2 Ω/sq and *T* = 86.9% with an FOM value of 142.8, which is competitive with most TCFs, including graphene, CNTs, PEDOT:PSS, CuNWs, and ITO. Although the AgNWs-based TCFs can reach higher FOM values due to their intrinsic electrical conductivity, the expensive price of silver restricts their industrial applications. Herein, we calculated the cost of the preparation of the CuNWs and AgNWs based on this study and our previous work [19], and the results clearly show that the CuNWs are 86.8% cheaper, mainly due to the lower cost of solvent and metal precursor, as shown in Fig. 6b. In addition, the aqueous process of preparing CuNWs is much more environmentally friendly than that of AgNWs using ethylene glycol as a solution, where toxicity and flammability are the most concerning issues [71]. The results show that the fabricated h-rGO/CuNWs TCF film possesses exceptional optoelectronic properties with high flexibility (Fig. 7), atmospheric oxidation resistance, and cost-effectiveness.

## Conclusions

In this study, we demonstrate a green approach to prepare h-rGO/CuNWs TCFs that possess good optoelectronic properties (*R*_sh_ = 25.1 Ω/sq and *T* = 85.9%), and the sheet resistance only slightly increased to 42.2 Ω/sq after 30 days. In addition, the uniformly covered h-rGO not only serves as a protection layer from oxidation but also increases the adhesion of CuNWs on the substrate due to the interaction between the functional groups on GO and CuNWs. The *R*_sh_ changed by only 1.0 Ω/sq after 2500 bending cycles at a bending radius of 5.3 mm, indicating the exceptional flexibility of the h-rGO/CuNWs film. Although the intrinsic electrical property of the CuNWs is less superior than that of the AgNWs, the benefits of CuNWs far outweigh the conductive loss, including inexpensiveness, better environmental friendliness, and nonflammability of the aqueous process. We believe that the h-rGO/CuNW TCF is a promising substitute material for ITO TCFs and can be used in various optoelectronic devices.

## Supplementary Information

**Additional file 1**: **Fig. S1**. SEM image of h-rGO/CuNWs with low magnification, indicating that CuNWs can be uniformly covered by h-rGO. **Fig. S2**. Raman spectra of GO, c-rGO, and h-rGO. The intensity ratio of the D and G bands (ID/IG ratio) increases from 0.99 for GO to 1.19 and 1.18 for c-rGO and h-rGO, respectively. **Fig. S3**. **a** AFM height images of the h-rGO/CuNWs TCF. **b** The plot is the height data of h-rGO/CuNWs pointed out by the blue dash line in the Fig S2a. The height difference between two rGO layers is around 3.4 nm, indicating a three-layered rGO structure. **Tab. S1**. Raman spectroscopy analyses of GO, rGO. and rGO coated with CuNWs samples. **Tab. S2**. Optoelectronic performances of some selected state-of-the-art flexible TCFs.

## Data Availability

The datasets used and/or analyzed during the current study are available from the corresponding author on reasonable request.

## Abbreviations

CuNWs: Copper nanowires
AgNWs: Silver nanowires
ITO: Tin-doped indium oxide
GO: Graphene oxide
c-rGO: Chemically reduced graphene oxide
h-rGO: Hydrogen-annealing reduced graphene oxide
TCF: Transparent conductive film
PET: Polyethylene terephthalate
HDA: Hexadecylamine
CVD: Chemical vapor deposition
IPA: Isopropyl alcohol
FOM: Figure of merit
SEM: Scanning electron microscopy
XRD: X-ray diffraction
*R*_sh_: Sheet resistance
*T*: Transmittance
