# Supplementary material for: A Green Approach for High Oxidation Resistance, Flexible Transparent Conductive Films Based on Reduced Graphene Oxide and Copper Nanowires
Source: Nanoscale Res Lett. 2022 Aug 24;17:79. doi: 10.1186/s11671-022-03716-1 (PMC9402884; doi:10.1186/s11671-022-03716-1)
Supplement: Supplementary file 1 — Additional file 1: Fig. S1. SEM image of h-rGO/CuNWs with low magnification, indicating that CuNWs can be uniformly covered by h-rGO. Fig. S2. Raman spectra of GO, c-rGO, and h-rGO. The intensity ratio of the D and G bands (ID/IG ratio) increases from 0.99 for GO to 1.19 and 1.18 for c-rGO and h-rGO, respectively. Fig. S3. a AFM height images of the h-rGO/CuNWs TCF. b The plot is the height data of h-rGO/CuNWs pointed out by the blue dash line in the Fig S2a. The height difference between two rGO layers is around 3.4 nm, indicating a three-layered rGO structure. Tab. S1. Raman spectroscopy analyses of GO, rGO. and rGO coated with CuNWs samples. Tab. S2. Optoelectronic performances of some selected state-of-the-art flexible TCFs. [file 11671_2022_3716_MOESM1_ESM.docx]

**Supporting Information**

**A Green Approach for High Oxidation-Resistance, Flexible Transparent Conductive Films Based on Reduced Graphene Oxide and Copper Nanowires**

Ya-Ting Lin^1^, Da-Wei Huang^2^, Pin-Feng Huang^2^, Li-Chun Chang^2^, Yi-Ting Lai^2,3,4^* and Nyan-Hwa Tai^1^*

^1^Department of Materials Science and Engineering, National Tsing Hua University, Hsinchu 30013, Taiwan.

^2^Department of Materials Engineering, Ming Chi University of Technology, New Taipei City 24301, Taiwan.

^3^Center for Plasma and Thin Film Technologies, Ming Chi University of Technology, Taiwan.

^4^Biochemical Technology R&D Center, Ming Chi University of Technology, Taiwan.

* Correspondence: laieating@mail.mcut.edu.tw; nhtai@mx.nthu.edu.tw

**Fig. S 1** SEM image of h-rGO/CuNWs with low magnification, indicating that CuNWs can be uniformly covered by h-rGO.

**Fig. S 2** Raman spectra of GO, c-rGO, and h-rGO. The intensity ratio of the D and G bands (I_D_/I_G_ ratio) increases from 0.99 for GO to 1.19 and 1.18 for c-rGO and h-rGO, respectively.

**Fig. S3** **a** AFM height images of the h-rGO/CuNWs TCF. **b** The plot is the height data of h-rGO/CuNWs pointed out by the blue dash line in the Fig S2a. The height difference between two rGO layers is around 3.4 nm, indicating a three-layered rGO structure.

**Tab. S 1** Raman spectroscopy analyses of GO, rGO. and rGO coated with CuNWs samples.

| Sample | D band (cm^-1^) | G band (cm^-1^) | I_D_/I_G_ ratio |
| --- | --- | --- | --- |
| GO | 1351 | 1603 | 0.99 |
| c-rGO | 1350 | 1593 | 1.19 |
| h-rGO | 1348 | 1590 | 1.18 |
| c-rGO/CuNWs | 1350 | 1598 | 1.18 |
| h-rGO/CuNWs | 1349 | 1600 | 1.18 |

**Tab. S 2** Optoelectronic performances of some selected state-of-the-art flexible TCFs.

| Materials | Substrate | Rs (Ω/sq) | *T* at 550 nm (%) | FOM | Ref. |
| --- | --- | --- | --- | --- | --- |
| Carbon materials | | | | | |
| Graphene | PET | 30 | 90.0 | 116.2 | [64] |
| Graphene | PET | 600 | 85.0 | 3.7 | [15] |
| Graphene | PET | 249 | 91.3 | 16.3 | [16] |
| rGO | PET | 800 | 82.9 | 2.4 | [1] |
| rGO | PET | 22000 | 88.0 | 0.1 | [28] |
| CNT | PI | 1169 | 77.6 | 1.2 | [14] |
| Conductive polymer | | | | | |
| PEDOT:PSS | PET | 57 | 87.0 | 45.9 | [18] |
| ITO | | | | | |
| ITO | PET | 48 | 83.0 | 40.2 | [65] |
| ITO | PET | 16 | 85.9 | 148.9 | [67] |
| Copper nanowires | | | | | |
| CuNWs | PET | 102.9 | 97.6 | 150.5 | [59] |
| CuNWs | PET | 30 | 83.0 | 64.4 | [60] |
| CuNWs | Chitosan composite | 14.1 | 83.7 | 143.7 | [61] |
| CuNWs | 3M tape | 20 | 80.0 | 79.8 | [62] |
| Silver nanowires | | | | | |
| AgNWs | PET | 19 | 88.0 | 150.3 | [66] |
| AgNWs | PET | 60 | 89.0 | 52.4 | [69] |
| AgNWs | PET | 20 | 93.0 | 255.0 | [19] |
| AgNWs | PET | 5 | 92.0 | 885.5 | [70] |
| Hybrid | | | | | |
| CuNWs/rGO | PET | 50 | 82.0 | 36.1 | [2] |
| CuNWs/EVA | PET | 48 | 91.0 | 81.3 | [40] |
| Cu/P4VP NWs | PET | 15.6 | 82.0 | 115.8 | [63] |
| rGO/PEDOT:PSS | PET | 51 | 82.0 | 35.4 | [29] |
| AgNWs/rGO | PET | 74 | 89.0 | 42.4 | [39] |
| AgNWs/PEDOT:PSS | PET | 75 | 92.0 | 59.0 | [68] |
| This work | | | | | |
| CuNWs/h-rGO | PET | 25.1 | 85.9 | 95.0 | This work |
| CuNWs/h-rGO | PET | 21.7 | 86.7 | 117.3 |  |
| CuNWs/h-rGO | PET | 18.2 | 86.9 | 142.8 |  |
|  | | | | | |
